# Graphite Whiskers Derived from Waste Coffee Grounds Treated at High Temperature

**DOI:** 10.1002/gch2.201800107

**Published:** 2019-02-12

**Authors:** Gan Jet Hong Melvin, Zhipeng Wang, Shingo Morimoto, Masatsugu Fujishige, Kenji Takeuchi, Yoshio Hashimoto, Morinobu Endo

**Affiliations:** ^1^ Material and Mineral Research Unit Faculty of Engineering Universiti Malaysia Sabah Jalan UMS 88400 Kota Kinabalu Sabah Malaysia; ^2^ Institute of Advanced Materials Jiangxi Normal University 99 Ziyang Avenue Nanchang Jiangxi 330022 China; ^3^ Institute of Carbon Science and Technology Shinshu University 4‐17‐1 Wakasato Nagano 380‐8553 Japan

**Keywords:** coffee grounds, graphite whiskers, high temperature, Raman spectroscopy

## Abstract

Graphite whiskers (GWs) are obtained from coffee grounds (CGs) treated at 2500 °C for 1 h in the presence of Ar gas at 1 atm. The majority of the GWs formed inside the CGs shell are rod‐like with a conical tip with diameter and length in the range between 1 to 3 µm and 4 to 10 µm, respectively. At first, the carbon layer might be grown in a turbostratic manner, and then progressively graphitized at higher temperature. The strong G′ peak intensity might be induced by the disclination of graphitized carbon layers.

Coffee grounds (CGs) can be categorized as agro‐based waste materials that are abundant in various countries, mainly produced from the by‐product of coffee beverage consumptions. Based on the report by Food and Agriculture Organization of the United Nations (FAO), world coffee production showed increasing trend from year 1994 to 2016 (≈9 million tons), which also reflect the high amount of CGs. Thus, it is important to conduct research on CGs, in order to fully utilize and unlock their potential as the alternative materials for broad applications. CGs have been modified for various usages such as soil remediation, adsorbent materials, and electrochemical energy devices.^1^, ^2^ In most of the cases, CGs need to be carbonized before their further utilization.

Up to today, several sources have been treated at high temperatures and they exhibited unique structures and morphologies. For instance, high purity and crystallinity of multilayer graphene can be obtained from rice husks treated at high temperature.^3^ Furthermore, sources such as fullerene waste soot,^4^ wood charcoal,^5^ and grounded graphite^6^ treated at high temperatures showed that graphite whiskers (GWs) can be formed. GWs that can be obtained from waste materials are interesting in the term to reutilize them in order to fabricate new products. In this study, the structure and Raman results of GWs obtained from CGs treated at high temperature will be reported. To the best of our knowledge, the Raman spectra of GWs obtained from CGs have not been examined.

First, the CGs were carbonized at 800 °C for 1 h under the presence of Ar gas, with heating rate 5 °C min^−1^. Then, they were treated at 2500 °C using high temperature graphite furnace under Ar gas flow for 1 h at 1 atm. The heating rate from room temperature to 2000 °C is 20 °C min^−1^, until 2500 °C is 5 °C min^−1^, and finally kept at 2500 °C for 1 h. Then, they were cooled down naturally, and their morphologies were observed using field emission scanning electron microscope (FE‐SEM; Hitachi SU‐8000) and transmission electron microscope (TEM; JEOL JEM‐2100F) with an accelerating voltage of 15 and 200 kV, respectively. Furthermore, Raman spectroscopy measurement was performed using RENISHAW inVia Raman spectroscope with 532 nm laser excitation.

FE‐SEM images of the GWs are depicted in **Figure** **1**. Notably, from Figure 1a,b, the CGs have cell structure and the GWs formed inside the cell. The majority of the GWs show two types of morphology: I) twisted GWs which roll up with its axial, and II) rod‐like GWs with a conical tip, as shown in Figure 1c,d. Furthermore, for the type II, most of them have smaller rod‐like shape at both ends. The diameter and length of GWs are in the range between 1 to 3 µm and 4 to 10 µm, respectively. FE‐SEM images of CGs treated at different temperatures and period are provided in Figure S1 in the Supporting Information.

**Figure 1:**
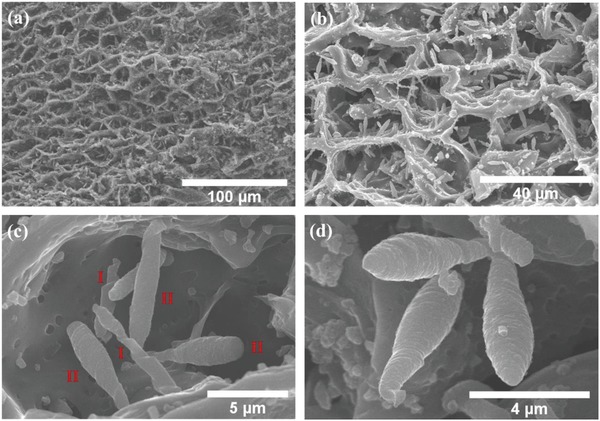
FE‐SEM images of a,b) low magnification and c,d) high magnification of GWs obtained from 2500 °C heat treatment for 1 h in the presence of Ar gas at 1 atm.

TEM images of the GWs are presented in **Figure** **2**. From Figure 2a,b, type II GWs, with different tip, conical and triangle‐like were observed, respectively. The observed planar distance, *d* = 0.345 nm, is slightly larger than (002) plane of single crystal graphite (*d* = 0.335 nm). Larger *d* was also observed in ref. 5, indicating that our GWs are turbostratic carbon. It is worth noticing, from Figure S2 in the Supporting Information, gibbous speckles were observed on the surface of GWs, further confirming the existence of protrusion or dangling bonds.^4^, ^6^ Other GWs (Figure 2d) showed loop‐like or frill‐like morphologies on their brims, showing the dangling bonds (free edge) reduction and no further growth (columnar), so that the GWs possess stable structure.^4^, ^5^ At first, the carbon layer might be grown in turbostratic manner, and then progressively graphitized at higher temperature.^6^ The apex angle was 143° (Figure 2c), in the range as reported in ref. 5 which the graphite layer formed a certain angle in the axis direction, dissimilar with those reported in refs. 7, 8 where the graphite layers scrolled up into cylindrical structure along the whisker axis and the filaments stacked perpendicular to their axis, respectively. From Figure 2c and Figure S3a in the Supporting Information, disclination (red rectangle box) which is represented by ripple‐like structure also can be observed. This might be attributed to the shrinkage of graphitized carbon layers, local strain, and minor orientation difference.^6^

**Figure 2:**
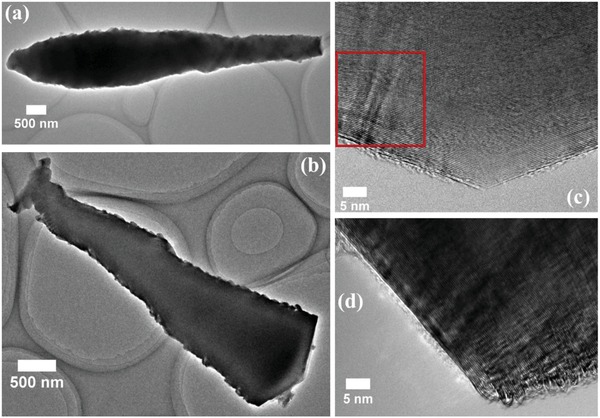
TEM images of GWs, a) conical tip, b) triangle‐like tip, c) ripple‐like structures, and d) loop‐like or frill‐like morphologies on brims.

Based on the collected data, a growth model is proposed. From Figure S4 in the Supporting Information, carbon and oxygen were traced from untreated CGs, and only carbon with low oxygen was traced from heat‐treated CGs. This indicated that the growth of our GWs is not related to other materials or catalysts. When the CGs are treated at high temperature, vaporized carbon can be assumed as the carbon source. Furthermore, from Figure S3b in the Supporting Information, graphitized and polyhedral particles can be spotted, which can be assumed as the nucleation sites during progressive heat treatment.^4^ As the GWs only can be noticed inside the cells, vaporized carbon became supersaturated inside the cells, in which the cells act as the reactor.^5^ Then, the vaporized carbon gradually deposited on the nucleation sites inside the cells, and the GWs are produced.

Raman spectroscopy is an effective tool to characterize carbonaceous materials. Our GWs are big enough to be characterized as depicted in **Figure** **3**a, and their Raman spectra are illustrated in Figure 3b,c. Inset of Figure 3b shows the Raman spectra of GW1‐A, 1200–1800 cm^−1^, where clearer D′ peak can be observed. The Raman spectra for other carbon materials such as single‐layer graphene (SLG), pyrolytic graphite sheet (PGS), and CG Area (area of carbonized CGs without GWs) are shown in Figure 3d. The details of D (≈1350 cm^−1^, requires defect for activation), G (≈1580 cm^−1^), D′ (≈1620 cm^−1^), and G′ (≈2700 cm^−1^, D overtone, requires no defect for activation) peaks position, full width at half maximum (FWHM), and ratios are tabulated in **Table** **1**. The significant characteristic of our GWs is the strong single Lorentzian G′ peak in contrast to the D or G peaks. It is worth noticing that the strong G′ intensity and the high *I*_G′_ */I*_G_ are not caused by the loop/frill‐like brims.^4^, ^9^ However, the circular Brillouin zone (BZ) can be expected based on the disclination (ripple‐like structure) and continuous rotational structure of GWs, instead of the hexagonal BZ of crystalline graphite, which stimulated the strong G′ intensity and the high *I*_G′_ */I*_G_.^4^, ^9^ Furthermore, the whole Raman spectra of GWs is clearly different from the SLG and PGS, where the D peak is not evident and the G′ intensity is not as strong as GWs for both (Figure 3d), and the G′ peak for PGS presented a shoulder (inset Figure 3d). The GWs exhibited clear D peak and the G′ peak upshifted around ≈20 cm^−1^ compared to SLG, high similarity with the turbostratic graphite with a single G′ peak.^10^ The Raman spectra between GWs and CG area also can be easily distinguished. The G′ peak FWHM for GWs is around 28–30 cm^−1^, agreeable range with ref. 4. Overall, the Raman spectra of all GWs showed high similarity, regardless of the different shape and size of them, and they are well graphitized with lower *I*_D_ */I*_G_ than the CG area. The *I*_G′_ */I*_D_ is around 10–13, slightly lower than ref. 4. This is due to the fact that more defects can be expected in our GWs, based on the obvious D and D′ peaks (structural defects or open edges).^2^, ^10^

**Figure 3:**
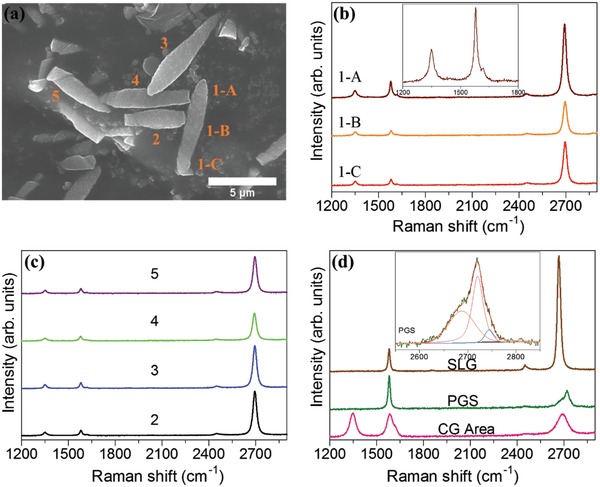
a) FE‐SEM image shows the GWs selected for Raman measurement, b,c) Raman spectra of GWs and d) Raman spectra of other carbon materials.

**Table 1:** Raman information of GWs, CG area, PGS, and SLG based on Figure 3

Sample	D band [cm ^−1^ ] Peak FWHM		G band [cm ^−1^ ] Peak FWHM		D′ band [cm ^−1^ ] Peak FWHM		G′ band [cm ^−1^ ] Peak FWHM		*I*_D_ */I*_G_	*I*_G′_ */I*_G_	*I*_G′_ */I*_D_
GW1‐A	1348.8	26.4	1577.8	16.9	1617.0	21.0	2691.6	27.8	0.67	6.91	10.25
GW1‐B	1350.2	23.7	1579.2	18.2	1618.0	20.7	2694.6	29.4	0.92	10.24	11.07
GW1‐C	1350.3	22.4	1580.0	16.6	1619.3	13.0	2694.6	29.4	0.93	11.7	12.55
GW2	1350.7	25.0	1579.8	17.3	1618.6	17.4	2694.3	29.4	0.91	12.68	13.95
GW3	1350.4	22.0	1579.6	17.4	1620.0	18.3	2693.9	30.3	0.82	10.63	13.04
GW4	1348.9	28.7	1578.0	18.3	1615.0	21.0	2691.9	30.2	0.94	9.08	9.63
GW5	1350.5	22.9	1580.1	17.3	1619.6	9.7	2694.8	29.6	0.83	11.08	13.40
CG area	1347.1	47.9	1584.2	44.5	1622.0	25.7	2690.0	78.4	1.14	1.52	1.34
PGS	‐	‐	1580.2	15.9	‐	‐	2720.5	27.0	‐	0.61	‐
SLG	‐	‐	1579.5	14.6	‐	‐	2668.2	26.0	‐	7.7	‐

In brief, GWs were synthesized from CGs treated at high temperature in the presence of Ar gas at 1 atm. The shrinkage of graphitized carbon layers, local strain, and minor orientation difference might cause the ripple‐like structure observed. The important feature of our GWs is the strong single G′ peak intensity and high *I*_G′_ */I*_G_, which might be attributed to the disclination of graphitized carbon layer.

## Conflict of Interest

The authors declare no conflict of interest.

## Supporting information

Supplementary

## References

[1] A. Reffas , V. Bernardet , B. David , L. Reinert , M. Bencheikh Lehocine , M. Dubois , N. Batisse , L. Duclaux , J. Hazard. Mater. 2010, 175, 779. 19942347 10.1016/j.jhazmat.2009.10.076

[2] Z. Wang , H. Ogata , S. Morimoto , M. Fujishige , K. Takeuchi , H. Muramatsu , T. Hayashi , J. Ortiz‐Medina , M. Z. M. Yusop , M. Tanemura , M. Terrones , Y. Hashimoto , M. Endo , J. Mater. Chem. A 2015, 3, 14545.

[3] G. J. H. Melvin , Z. Wang , N. J. Siambun , M. M. Rahman , IOP Conf. Ser.: Mater. Sci. Eng. 2017, 217, 012017.

[4] Z. Wang , H. Ogata , S. Morimoto , M. Fujishige , K. Takeuchi , Y. Hashimoto , M. Endo , Carbon 2015, 90, 154.

[5] Y. Saito , T. Arima , Carbon 2007, 45, 248.

[6] J. Dong , W. Shen , B. Zhang , X. Liu , F. Kang , J. Gu , D. Li , N. Chen , Carbon 2001, 39, 2325.

[7] R. Bacon , J. Appl. Phys. 1960, 31, 283.

[8] H. Murayama , T. Maeda , Nature 1990, 345, 791.

[9] J. Dong , W. Shen , B. Tatarchuk , Appl. Phys. Lett. 2002, 80, 3733.

[10] A. C. Ferrari , D. M. Basko , Nat. Nanotechnol. 2013, 8, 235. 23552117 10.1038/nnano.2013.46

