# Supplementary material for: Graphite Whiskers Derived from Waste Coffee Grounds Treated at High Temperature
Source: Glob Chall. 2019 Feb 12;3(8):1800107. doi: 10.1002/gch2.201800107 (PMC6686167; doi:10.1002/gch2.201800107)
Supplement: Supplementary file 1 — Supplementary [file GCH2-3-1800107-s001.pdf]

Copyright WILEY-VCH Verlag GmbH & Co. KGaA, 69469 Weinheim, Germany, 2019.

Global Challenges
Open Access

Supporting Information

for *Global Challenges,* DOI: 10.1002/gch2.201800107

Graphite Whiskers Derived from Waste Coffee Grounds Treated at High Temperature

Gan Jet Hong Melvin, Zhipeng Wang, Shingo Morimoto, Masatsugu Fujishige, Kenji Takeuchi, Yoshio Hashimoto, and Morinobu Endo*

Copyright WILEY-VCH Verlag GmbH & Co. KGaA, 69469 Weinheim, Germany, 2019.

Supporting Information

Graphite Whiskers Derived from Waste Coffee Grounds Treated at High Temperature

Gan Jet Hong Melvin*, Zhipeng Wang, Shingo Morimoto, Masatsugu Fujishige, Kenji Takeuchi, Yoshio Hashimoto, and Morinobu Endo

The GWs were not formed at 1500°C and 1800°C, as shown in Figure S1(a) and (b). Furthermore, from Figure S1(c) and (d), suspected not fully grown GWs or precursor of GWs were observed at 2000°C, all over inside the carbonized CGs cell, indicating that temperature over 2000°C will promote the growth of GWs. Moreover, less or partially GWs were observed when the CGs are treated at 2500°C for 0.5h as shown in Figure S1(e) and (f), in comparison to treatment at 2500°C for 1h (Figure 1(a)).

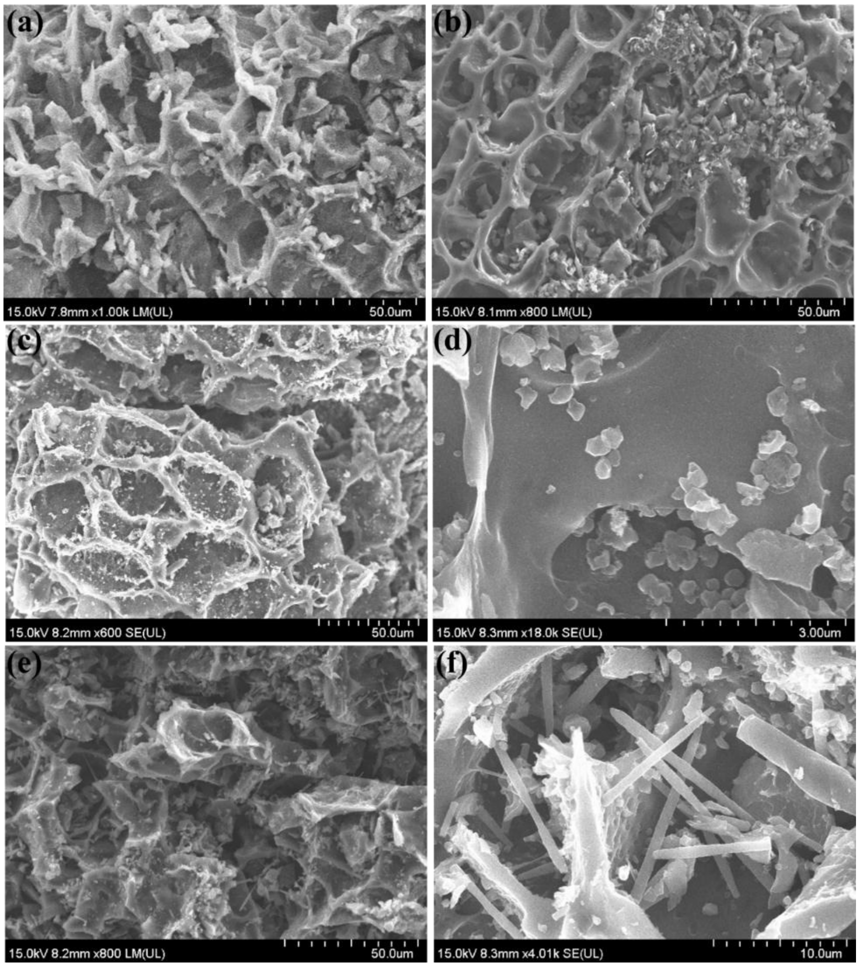

Figure S1. FE-SEM images of CGs treated at various temperatures for 0.5 h (a) 1500°C, (b) 1800°C, (c, d) 2000°C and (e, f) 2500°C.

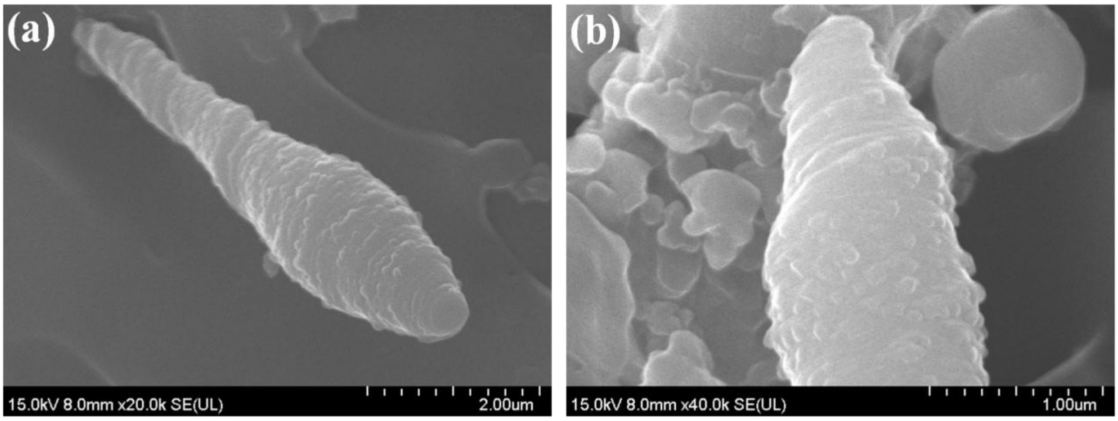

Figure S2. (a, b) FE-SEM images of CGs treated at 2500°C with gibbous speckles.

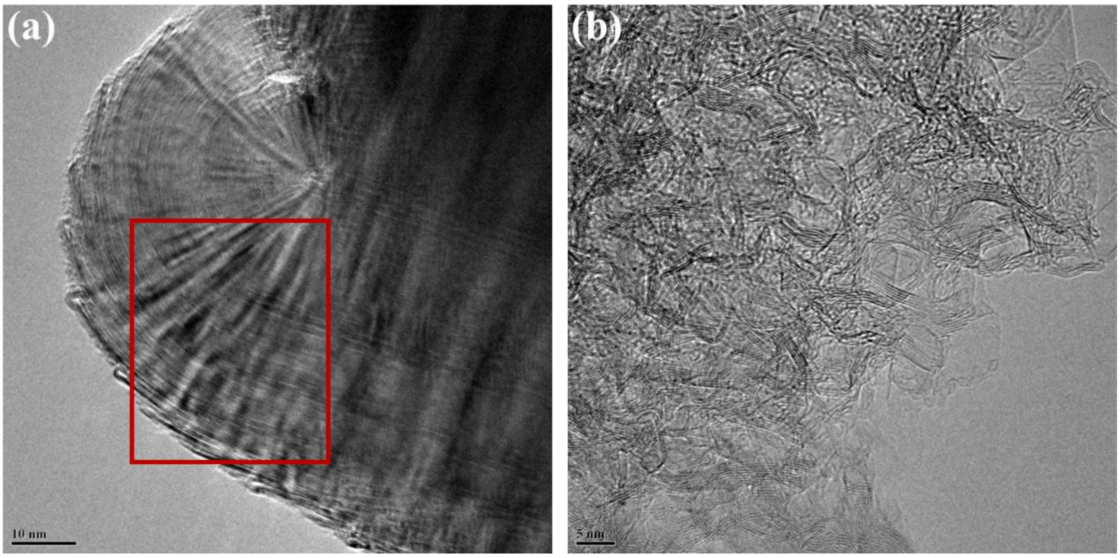

Figure S3. TEM images of (a) disclination and (b) graphitized particles.

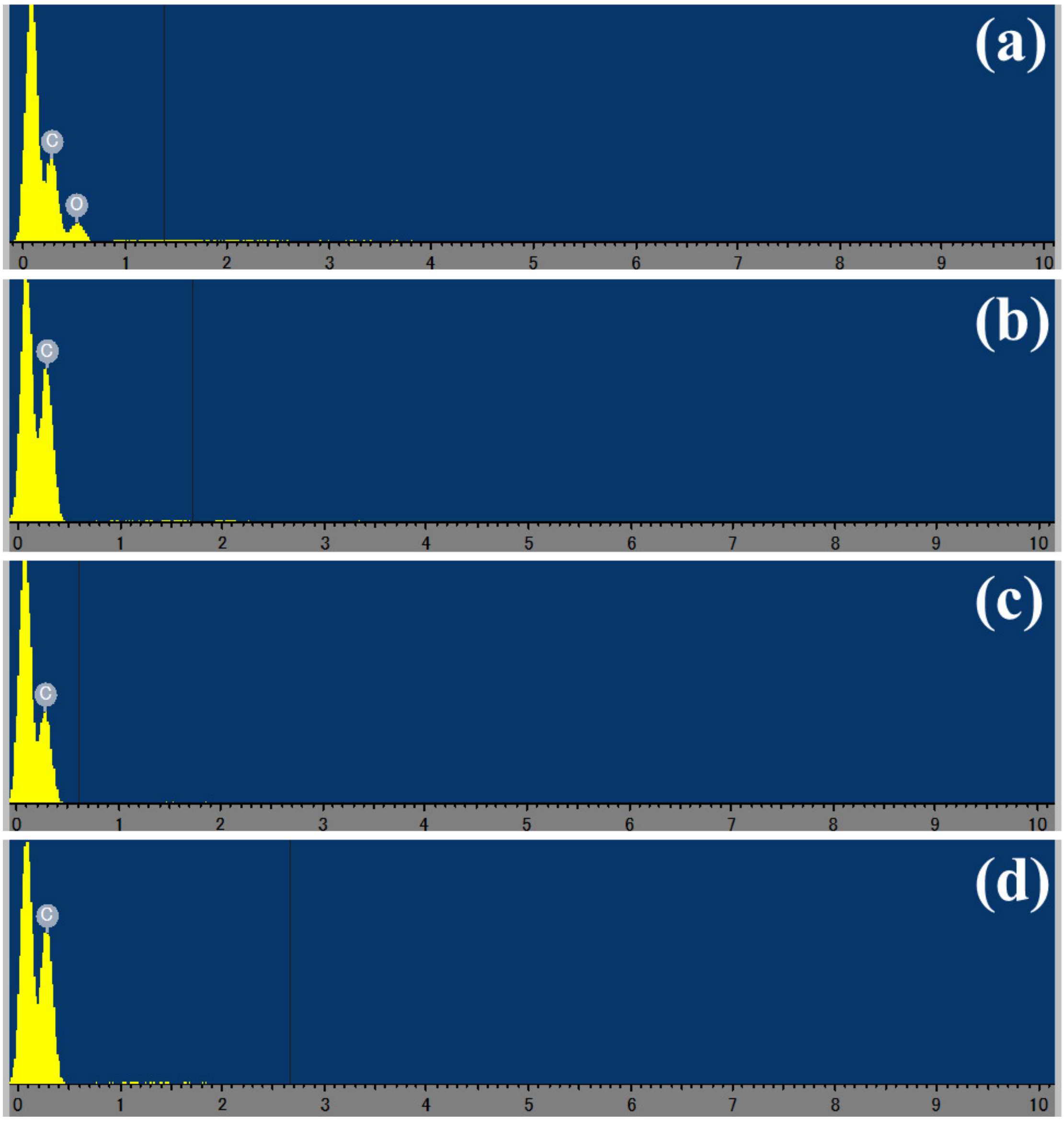

Figure S4. EDX of CGs treated at various temperatures (a) pristine, (b) 1500°C, (c) 2000°C and (d) 2500°C.
